# Noninvasive ventilation improves cardiac function in patients with chronic heart failure

**DOI:** 10.18632/oncotarget.10441

**Published:** 2016-07-06

**Authors:** Jing Cheng, Yanping Liu, Guishuang Li, Zhongwen Zhang, Lianyue Ma, Xiaoyan Yang, Jianmin Yang, Kai Zhang, Jing Kong, Mei Dong, Meng Zhang, Xingli Xu, Wenhai Sui, Jiali Wang, Rui Shang, Xiaoping Ji, Yun Zhang, Cheng Zhang, Panpan Hao

**Affiliations:** ^1^ The Key Laboratory of Cardiovascular Remodeling and Function Research, Chinese Ministry of Education and Chinese Ministry of Health, Shandong University Qilu Hospital, Jinan, Shandong, China; ^2^ Shandong Key Laboratory of Cardiovascular and Cerebrovascular Disease, Shandong Provincial Medical Imaging Institute, Shandong University, Jinan, Shandong, China; ^3^ Shandong Provincial Qianfoshan Hospital, Shandong University, Jinan, Shandong, China

**Keywords:** noninvasive ventilation, chronic heart failure, sleep-disordered breathing, left ventricular ejection fraction, brain natriuretic peptide, Pathology Section

## Abstract

Chronic heart failure (CHF) has been shown to be associated with an increased incidence of sleep-disordered breathing. Whether treatment with noninvasivepositive-pressure ventilation (NPPV), including continuous positive airway pressure, bi-level positive airway pressure and adaptive servo-ventilation, improves clinical outcomes of CHF patients is still debated. 2,832 CHF patients were enrolled in our analysis. NPPV was significantly associated with improvement in left ventricular ejection fraction (39.39% vs. 34.24%; WMD, 5.06; 95% CI, 3.30-6.81; *P* < 0.00001) and plasma brain natriuretic peptide level (268.23 pg/ml vs. 455.55 pg/ml; WMD, −105.66; 95% CI, [−169.19]-[−42.13]; *P* = 0.001). However, NPPV did not reduce all-cause mortality (0.26% vs. 0.24%; OR, 1.13; 95% CI, 0.93-1.37; *P* = 0.22) or re-hospitalization rate (57.86% vs. 59.38%; OR, 0.47; 95% CI, 0.19-1.19; *P* = 0.02) as compared with conventional therapy. Despite no benefits on hard endpoints, NPPV may improve cardiac function of CHF patients. These data highlight the important role of NPPV in the therapy of CHF.

## INTRODUCTION

Sleep-disordered breathing (SDB), including Cheyne-Stokes respiration with central sleep apnea (CSR-CSA) and obstructive sleep apnea (OSA), is highly prevalent in patients with chronic heart failure (CHF). Large scale studies revealed that the prevalence of SDB in CHF patients is about 69% ~76% [1]. CHF patients with CSA might be suffering from greater augmented sympathetic nervous activity (SNA) than those without CSA [2]. Activated SNA increases peripheral vascular resistance and cardiac load, thus, exacerbating prognosis and remodeling cardiac structure. SDB is usually treated with noninvasive positive pressure ventilation (NPPV), which includes continuous positive airways pressure (CPAP), bi-level positive airway pressure (Bi-PAP) and adaptive servo-ventilation (ASV). NPPV can improve respiratory disturbances to different degree, and thereafter reduce venous return, cardiac preload and pulmonary congestion [3–5]. NPPV has been recommended and widely used in the respiratory management of patients with acute heart failure (AHF) [6, 7]. Although NPPV has been confirmed to improve the hemodynamics in AHF inpatients, this therapy in CHF outpatients was controversial [8, 9].

Drug therapy, mainly including angiotensin-converting enzyme (ACE) inhibitors, angiotensin II receptor blockers (ARBs) and β-blockers, has substantially improved the prognosis of patients with mild to moderate CHF, but the prognosis of patients with severe CHF remains poor. Hence, novel nonpharmacotherapy such as NPPV that might extend life expectancy and improve quality of life of CHF patients is required. However, studies focusing on effects of NPPV on CHF prognosis were limited by small sample size and inconclusive. Thus, we used powerful pooled-analysis methodology to investigate the impact of NPPV on all-cause mortality, re-hospitality and cardiac function in CHF patients, regardless of breathing status during sleeping.

## RESULTS

We identified 8,869 potentially eligible literature citations, of which 56 were reviewed as full articles. Only 23 reports of studies with suitable data were eligible for inclusion, all of which were full articles published in English from 1997 to 2015 [3, 10–31]. A total of 2,832 subjects were enrolled in the pooled analysis, of which 2,408 were from 15 randomized control trials (RCTs) [10–24] and 424 from 8 observational studies [3, 25–31]. The interobserver agreement for the study selection was excellent (κ = 0.91).

The sample sizes ranged from 14 to 1,325 participants. The mean ages of the study participants ranged from 56.9 to 72.8 years and the mean follow-up from 1 to 24 months. The methodological quality of the included studies was in general poor, with only 9 of 23 reports describing two or more of the four quality criteria. Loss to follow-up was reported as < 10% in only 16 reports.

### Clinical end-points

Cardiovascular events as well as cardiac function were evaluated for all 23 studies (Table 1). Although NPPV did not differ from conventional therapy in reducing all-cause mortality (0.26% *vs*. 0.24%; pooled odds ratio [OR], 1.13; 95% confidence interval [CI], 0.93-1.37; *P* = 0.22) or re-hospitality (57.86% *vs*. 59.38%; OR, 0.47; 95% CI, 0.19-1.19; *P* = 0.02), it significantly reduced left ventricular ejection fraction (LVEF) (39.39% *vs*. 34.24%) with a pooled weighted mean difference (WMD) of 5.06 (95% CI, 3.30-6.81; *P* < 0.00001) and plasma brain natriuretic peptide (BNP) level (268.23 pg/ml *vs*. 455.55 pg/ml) with a WMD of −105.66 (95% CI, [−169.19]-[−42.13]; *P* = 0.001) as compared with conventional therapy in CHF patients.

**Table 1:** Results of clinical events and cardiac function

Outcomes	References	Patients	OR/WMD (95% CI)	*P* value	I^2^, %	Heterogeneity *P* value
Mortality	10,11,15-17,19,23,27,31	2311	1.13 [0.93,1.37]	*P* = 0.22	48%	*P* = 0.05
Re-hospitalization rate	14-16,31	1612	0.47 [0.19,1.19]	*P* = 011	68%	*P* = 0.02
LVEF	3,10,12,14,15,18-22,24-26,28-31	785	5.06 [3.30,6.81]	*P* < 0.00001	82%	*P* < 0.00001
BNP	3,15,30,31	367	−105.66 [−169.19, −42.13]	*P* = 0.001	34%	*P* = 0.21

BNP, brain natriuretic peptide; CI, confidence interval; LVEF, left ventricular ejection fraction; OR, odds ratio; WMD, weighted mean difference.

### Sensitivity/subgroup analysis

For the sensitivity analysis, removal of the largest study [16] produced no significant alterations in pooled WMDs/ORs, indicating that the results of our pooled analysis were statistically reliable.

Heterogeneity was addressed well in the analyses of all-cause mortality (*I^2^* = 48%) and plasma BNP level (*I^2^* = 34%). However, high heterogeneity existed in the analyses of LVEF (*I^2^* = 82%) and re-hospitality (*I^2^* = 68%). After removal of one study [24] from the analysis of LVEF, the heterogeneity was significantly decreased to 30% (WMD 3.59, favoring NPPV; 95% CI, 2.47-4.70; *P* < 0.00001). Thereafter, we performed subgroup analyses in terms of LVEF (Table 2). The between-study heterogeneity was explained in part by the variability in concomitant breathing status during sleeping, NPPV subtypes and follow-up duration, but not by study design or race.

### Fail-safe number (Nfs)

We calculated the Nfs0.05 for each comparison and found the N_fs0.05_ values for all-cause mortality, re-hospitality, LVEF and plasma BNP to be greater than the numbers of studies included in the analyses. However, the N_fs0.05_ value for cardiovascular death was smaller than the number of retrieved studies, which was possibly consistent with small-study-related bias.

**Table 2:** Subgroup analyses with regard to the risk of LVEF

Subgroup	Studies	Patients, *n*	WMD (95% CI)	*P* value	I^2^,%	Heterogeneity *P* value
Adjustment for study design						
RCTs	10,14,15,18-22,24,25,28	567	4.13 [2.01,6.24]	*P* < 0.00001	82%	*P* < 0.00001
Observational studies	3,12,26,29-31	218	6.84 [3.11,10.57]	*P* = 0.0002	80%	*P* < 0.0004
Adjustment for race						
Caucasian	10,19-22,24-26,28,29	345	4.36 [2.34,6.38]	*P* < 0.00001	84%	*P* < 0.00001
Asian	3,12,14,15,18,30,31	440	6.43 [2.41,10.45]	*P* < 0.002	77%	*P* < 0.0003
Adjustment for breathing status during sleeping						
CSR-CSA	14,19,26,29-31	167	6.68 [2.54,10.82]	*P* = 0.002	79%	*P* = 0.0002
OSA	22,24,25,28	110	6.68 [5.20,8.16]	*P* < 0.00001	13%	*P* = 0.33
SDB	3,10,12,18,20,21	303	3.03 [1.12,4.94]	*P* = 0.002	35%	*P* = 0.17
Unclear	15	205	1.70 [−1.27,4.67]	*P* = 0.26	NA	NA
Adjustment for NPPV subtypes						
CPAP	21,22,24-26,28,29	211	3.56 [3.01,4.10]	*P* < 0.00001	88%	*P* < 0.00001
Bi-PAP	30	14	13.50 [9.71,17.29]	*P* < 0.00001		NA
ASV	3,10,12,14,15,18-20,31	560	3.06 [1.39,4.74]	*P* = 0.0003	3%	*P* = 0.41
Adjustment for follow-up duration						
> 3 month	3,14,15,18,31	411	3.48 [1.45,5.52]	*P* = 0.0008	15%	*P* = 0.32
≤3 month	10,12,19-22,24-26,28,30	374	3.71 [3.18,4.24]	*P* < 0.00001	87%	*P* < 0.00001

ASV, adaptive servo-ventilation; Bi-PAP, bi-level positive airway pressure; CI, confidence interval; CPAP, continuous positive airways pressure; CSR-CSA, Cheyne–Stokes respiration with central sleep apnea; LVEF, left ventricular ejection fraction; OSA, obstructive sleep apnea; SDB: Sleep-disordered breathing; RCTs, randomized controlled trials; WMD, weighted mean difference.

## DISCUSSION

The results indicated that NPPV was more efficacious in decreasing LVEF and plasma BNP level than conventional therapy in CHF outpatients, though it did not exhibit significant effect on the all-cause death and re-hospitalization. The pathophysiological features of this effect remain to be elucidated.

Stable CHF has been shown to be associated with an increased incidence of SDB, especially CSA. CHF patients develop CSA during both wakefulness and sleep, which typically demonstrate baseline hypocapnia [32, 33]. Hypocapnia from hyperventilation may be due to chronic interstitial pulmonary congestion, stimulating the pulmonary vagal reflex [34] or from the hypoxemic ventilatory response [35]. The coexistence of SDB and CHF is associated with poor survival. CSA decreases oxygen saturation and increases muscular SNA in CHF patients [36]. This additional stimulus to sympathetic nerve may accelerate the progression of CHF. A wealth of evidence has demonstrated that inhibition of a hyperactive SNA by ACE inhibitors, ARBs or β-blockers protects against LV remodeling and heart failure. Furthermore, we have found that ACE2 and Ang-(1-7), 2 new components of renin-angiotensin system, mitigate diabetic cardiomyopathy [37–39]. Sympathetic-inhibitory effect of NPPV might play a critical role in the treatment of CHF.

With respect to OSA, negative intra-thoracic pressure is exaggerated by inspiratory efforts against the occluded pharynx. It increases not only the right ventricle preload but also the right ventricular afterload. Negative intrathoracic pressure could increase death caused by cardiovascular events [40, 41]. CPAP has been reported to reduce the severity of SDB, improve exercise capacity, and improve cardiac function [11, 12, 42]. Effective CPAP reduced respiratory disturbances and cardiac haemodynamic parameters such as systolic/diastolic blood pressure. Furthermore, it improved cardiovascular neurohumoral function, sympathetic markers and quality of life [43]. It is well recognized that ASV is described as the most effective alternative for CSR-CSA in CHF patients [25]. A recent meta-analysis showed that chronic ASV therapy improved cardiac function in CHF with SDB [44]. Our data indicated NPPV therapy mitigated cardiac dysfunction in CHF patients, regardless of breathing status during sleeping. NPPV may improve cardiac function through increasing oxygen saturation, as well as decreasing SNA and blood pressure.

NPPV is a well established and increasingly used therapeutic option to treat patients with chronic hypercapnic respiratory failure that arises from different etiologies [45]. In cardio-respiratory disorders, NPPV is commonly used in treatments of AHF, chronic obstructive pulmonary disease, obesity hypoventilation syndrome, cystic fibrosis and neuromuscular disorders. Nowadays, various modes of NPPV have been investigated to treat CHF. The initial use of NPPV is CPAP, addressing the common component of increased upper airway resistance and obstruction. It also provide additional benefits of blood pressure reduction and decreased cardiac transmural pressure, thereby decreasing preload and afterload and improving LVEF [46]. However, patients using CPAP arise some side effects. Some studies showed potential hypovolemic effect with CPAP by decreased filling pressures in such patients [47, 48]. Bi-PAP in patients with CSR-CSA and CHF might improve apnea hypoventilation index, sleep quality and LVEF compared with Non-PAP or CPAP [26, 49, 50]. ASV is designed to average antecedent direction, magnitude and rate of change of airflow, providing inspiratory positive airway pressure support that varies dynamically according to the sensed patient airflow, essentially resulting in complementary ventilatory support appropriate to the patients' alternating hyperpneas and hypopneas. ASV use in CHF patients may improve physiologic parameters and reduce adverse cardiovascular events [13]. Even, ASV has also been used to treat CSR-CSA in CHF patients with preserved ejection fraction [51]. ASV significantly reduced the severity of sleep apnea, especially producing a better elimination of CSA than CPAP, and counteracted hypoxia to prevent subsequent inflammatory responses [52].

Several limitations should be considered in interpreting our results. First, high heterogeneity existed in analyses of re-hospitalization rate and LVEF. The pooled analysis had significant disparity in sample sizes of the studies considered. In fact, among the 2,952 patients included in the analysis, 1,325 (44.9%) were from Cowie et al study [14]. However, after removing data of this study, we found nosubstantial alteration in pooled ORs/WMDs. Second, the sample sizes of some studies were small and the smallest study only enrolled 14 patients [26]. However, the decision to include small-sample studies was necessary to maximize the utilization of all available data on this important topic. Third, the different results between hard endpoints (death and rehospitalization) and surrogate endpoints (BNP and LVEF) might be attributed to the relatively short follow-up durations. The follow-ups were relatively short, and that was 24 months in one study, 12 months in 3 studies, 6 months in 5 studies and < 6 months in the other studies. Thus, more large-scale, multinational, multicenter, randomized, controlled and long term follow-up trials are warranted.

## MATERIALS AND METHODS

### Data sources, search strategy, and selection criteria

We performed a systematic computerized search of MEDLINE, EMBASE, Web of science and Cochrane databases to identify clinical trials assessing effects of NPPV treatments (CPAP, Bi-PAP, and ASV) on clinical outcomes in CHF patients. Bibliographies of retrieved articles, previous pooled analysis, and reviews were also used to identify any potentially relevant publications miss in our search. We had no restriction on language or whether the results had been published.

Literatures were searched in MEDLINE with the terms (“ASV” or “adaptive servo ventilation” or “adaptive ventilation” or “servoventilation” or “Bi-PAP” or “bi-level positive airway pressure” or “CPAP” or “continuous positive airway pressure” or “noninvasive ventilation” [MeSH Terms] AND (“heart failure” or “chronic heart failure”) with no restriction on subheadings (up to 24 March, 2016). Similar but adapted search terms were used for the other literature databases or search engines. In order to increase sample size, widen population coverage and increase statistical power, we included results for both RCTs and observational studies.

The study selection criteria were as follows: (i) compared NPPV treatment (CPAP, Bi-PAP or ASV) *vs*. control group without NPPV in patients with diagnosis of stable CHF; (ii) were RCTs or observational studies with at least a 1-month follow-up for each group; and (iii) contained sufficient information to estimate the pooled WMDs or the pooled ORs and their corresponding 95% CIs.

### Data extraction

The following information was extracted from published reports by use of a standardized protocol: first author's last name, year of publication, study design, country of origin, number of enrolled patients, subject characteristics at baseline (age, sex, LVEF, family history of cardiovascular disease, NYHA Class), follow-up duration, concomitant medication, and end-points.

### Quality assessment

The methodological quality of eligible studies was assessed with criteria adapted from guidelines for the evaluation of articles on prognosis [53]. he literature search, data extraction and quality assessment were undertaken independently and blindly by two authors (J.C. and Y.P.L.) using a standardized approach. Any disagreements were resolved by a third reviewer (P.P.H.).

### Statistical analysis

RevMan 5.3 software, developed by the Cochrane Collaboration (http://www.cc-ims.net/revman, accessed on 16 November 2015) was used for the pooled analysis. The between-study heterogeneity was tested by the chi-square-based Cochran's Q statistic and the inconsistency index (I^2^). Statistically significant heterogeneity was considered with a chi-square *P* < 0.10 and *I^2^* > 50%. Results showing no significant heterogeneity were analyzed by the fixed-effects model and those with significant heterogeneity were analyzed by the random-effects model. Pooled WMDs or ORs were reported with 95% CIs, and a two-tailed *P* < 0.05 was considered statistically significant for all analyses. In addition, sensitivity or subgroup analyses were conducted to seek more narrowly drawn subsets of reports of studies by removing an individual study each time or studies with similar features to assess individual effects. Finally, we assessed publication bias using Nfs. Any calculated Nfs value smaller than the number of retrieved reports of studies indicated publication bias. We calculated the N_fs0.05_ as N_fs0.05_ = (ΣZ/1.64)^2^ - k, where k is the number of reports of studies included in the analysis.
